# Homeostasis in anorexia nervosa

**DOI:** 10.3389/fnins.2014.00234

**Published:** 2014-08-06

**Authors:** Per Södersten, Cecilia Bergh, Modjtaba Zandian, Ioannis Ioakimidis

**Affiliations:** Section of Applied Neuroendocrinology, Karolinska InstitutetHuddinge, Sweden

**Keywords:** body weight, physical activity, obesity, anorexia, orexigens, homeostasis

## Abstract

Brainstem and hypothalamic “orexigenic/anorexigenic” networks are thought to maintain body weight homeostasis in response to hormonal and metabolic feedback from peripheral sites. This approach has not been successful in managing over- and underweight patients. It is suggested that concept of homeostasis has been misinterpreted; rather than exerting control, the brain permits eating in proportion to the amount of physical activity necessary to obtain food. In support, animal experiments have shown that while a hypothalamic “orexigen” excites eating when food is abundant, it inhibits eating and stimulates foraging when food is in short supply. As the physical price of food approaches zero, eating and body weight increase without constraints. Conversely, in anorexia nervosa body weight is homeostatically regulated, the high level of physical activity in anorexia is displaced hoarding for food that keeps body weight constantly low. A treatment based on this point of view, providing patients with computerized mealtime support to re-establish normal eating behavior, has brought 75% of patients with eating disorders into remission, reduced the rate of relapse to 10%, and eliminated mortality.

## Introduction

The obvious goal of treatment of over- and underweight is to normalize the weight of the patients and restore their health. We offer an explanation why current methods fail, and we outline the rationale for an alternative approach and describe its outcome.

From our perspective, the reason for the limited success in managing body weight problems is because the concept of homeostasis as applied to the brain and eating behavior is a misinterpretation of the suggestion of Bernard (1865), the follow-up experiments of Cannon (1932), and the synthesis of Stellar (1954). In addition, most accounts of underweight patients do not even consider eating behavior (e.g., Boraska et al., 2014). Also, the cause of undesirable changes in body weight is thought to be related of a brain dysfunction, although there is no evidence that this is the case (Södersten et al., 2011).

Eating behavior has evolved to meet the challenge of starvation, an efficient foraging strategy has been encouraged, and satiety has been discouraged (Södersten et al., 2008; McCue, 2012). Further on, eating behavior mediates between the physical and mental state of the patient (Ioakimidis et al., 2011), and most neural, endocrine, and behavioral effects are reversible adaptations to the shortage of food, rather than signs of disease (Södersten et al., 2008).

## Overweight

Some aspects of overweight will be considered first, because overweight is on the extreme opposite of anorexia on the same behavioral and biochemical continuum (Bergh et al., 2008).

All levels of nearly all societies, from the individual to the systems of health care and finance, are affected by the problem of obesity. Human biology has been taken by surprise; we are not equipped with counter-regulatory mechanisms protecting us from the medical consequences of overeating and weight gain. These consequences have been reviewed many times and one conclusion stands out: most medical effects of obesity are reversible.

Pharmacological approaches have miniscule effects and unavoidable, undesirable side effects, which may include changes in mood and addiction (Poulton and Nanan, 2014; Yanovski and Yanovski, 2014). And although a minor decrease in body weight improves the patient's health, most patients relapse within a brief period of time (Yanovski and Yanovski, 2014). Not so with irreversible gastric surgery, which has a much bigger effect, the improvement of health is unquestionable (Carlsson et al., 2012). Alas, upon long-term follow-up, the patients may relapse into diabetic problems even though their weight may not return to its pre-operative level of obesity (Sjöström et al., 2014). Because surgeons cannot possibly operate on all the patients in need, it was recently suggested that gastric surgery might be replaced by the mediators of its effects, i.e., the release of bile acids and the emergent improvement of metabolic parameters (Ryan et al., 2014). Characteristically, the study ended in the exact same manner as all other studies on the topic of obesity by “suggesting new targets for … therapeutic interventions.” The same suggestion has emerged each time when the relatively rare obese genotypes have been discovered (Pearce et al., 2013). Sadly, most of these suggestions have failed (Troke et al., 2014).

## Underweight

The following description of the outcome in anorexia nervosa was published seven times the last year in the Lancet:

“Anorexia nervosa is characterized by a chronic course that is refractory to treatment in many patients and has one of the highest mortality rates of any psychiatric disorder,” and: “The evidence base for anorexia nervosa treatment is meager considering the extent to which this disorder erodes quality of life and takes far too many lives prematurely” (Lipsman et al., 2013; Treasure and Schmidt, 2013; Attia, 2014; Bulik, 2014; Editorial, 2014; Herpertz-Dahlmann et al., 2014; Zipfel et al., 2014). The same outcome was described 20 years earlier (Bergh and Södersten, 1998). Clearly, this field is at a standstill.

Conventional approaches to the management of anorexic patients rely on assumption that a chronic mental illness causes anorexia (Wierenga et al., 2014). This hypothesis persists although interventions targeting mental illness have no effect and despite the fact that the list of the other counter-arguments is very long (Bergh et al., 2013). While patients may go into remission from their anorexic symptoms, they remain symptomatic upon discharge from standard care, and the majority is reported to relapse within one year (Bergh et al., 2013). This is why anorexia is considered a chronic disease, associated with fatalistic views, e.g., co-morbidity, relapse, and mortality, which resist counterfactual scientific evidence (Dar-Nimrod and Heine, 2011).

An attempt at analyzing obesity in a psychiatric, neuroscience framework was unsuccessful, and therefore not pursued (Ziauddeen et al., 2012). By contrast, attempts at analyzing anorexia in the same manner are made repeatedly. The problem with these studies is that it is not possible to dissociate the state of the patient from her/his hypothetical mental illness (Wierenga et al., 2014). Patients may be in remission from anorexia, but the changes which are found in the brain may be related to the remaining symptoms, rather than the anorexic symptoms from which the patients are thought to have recovered (Wierenga et al., 2014). At present, there are no reports of brain function in anorexic patients who are in remission from *all* symptoms, although an excellent study came pretty close (Cowdrey et al., 2011).

Unlike the rare obese genotypes which have been found (e.g., Pearce et al., 2013), no anorexic genotype has been demonstrated. Hence the reticence in recent publications of the “possible role of known or putatively functional markers in genes regulating appetite and weight” in anorexia and bulimia, which “If replicated, … may serve as an important first step toward gaining a better understanding of weight regulation in eating disorders” (Yilmaz et al., 2014), and “Findings from candidate gene studies of [anorexia nervosa] resemble those for most complex biomedical diseases—initial intriguing findings diminished by the absence of rigorous replication” (Boraska et al., 2014).

We suggest that analyses of both obesity and anorexia fail because the biology of eating behavior is not taken into consideration.

## Homeostasis

Bernard (1865) first suggested the existence of internal counter-regulatory mechanisms protecting against external variation, keeping the internal milieu constant. Cannon (1932) developed Bernard's idea and coined the term “homeostasis” to designate “a condition that may vary, but which is relatively constant.” It is important to note that Cannon's experiments were based on the study of depletion (Södersten et al., 2008). Interestingly, however, he suggested that: “Cooperating with hunger and thirst in a way not yet clearly defined is the sensation of having had enough. Protection of the organism of being overstocked with food and water is thus obtained.” However, Cannon did not test the hypothesized satiety mechanism and if he had, he had very likely falsified it. In parallel, Stellar (1954) launched the dual center theory according to which eating behavior is controlled by the activity of anatomically separable centers in the hypothalamus which excite or inhibit eating as a result of feedback from peripheral endocrine and/or metabolic events. Stellar's (1954) theory has permeated all research in the area, although excitation and inhibition of eating are now thought to be chemically mediated by partially separable neural networks extending from the brainstem to the forebrain (Grill and Hayes, 2012).

However, obesity keeps increasing (Ng et al., 2014), providing compelling evidence against the existence of internal satiety mechanisms protecting us from being overstocked. Instead, it was recently pointed out that eating too much is a normal response to a change in the environment, i.e., the availability of energy-dense foods at a low economic and physical price (Swinburn et al., 2011). These external influences can be added to the concept of homeostasis as follows.

## The human homeostatic phenotype

Shortage of food is the main challenge in evolution and animals, including humans, have developed sophisticated strategies to forage for food (McCue, 2012). Addition of this decisive biological fact to the homeostatic framework has increased our understanding of how the brain is engaged in eating behavior. This analysis started by the observation that rats of both sexes can show consummatory ingestive and sexual behavior simultaneously (Kaplan et al., 1992). This should not surprise as these kinds of behavior engage different muscles. By contrast, appetitive behavior engages the same muscles independent upon what reward is going to be consumed. Interestingly, activation of appetitive sexual behavior blocks appetitive ingestive behavior (Kaplan et al., 1992). A series of experiments revealed that one hypothesized hypothalamic “orexigen,” neuropeptide tyrosine (NPY), exerts the opposite effect by selectively stimulating appetitive ingestive behavior at the expense of eating, and simultaneously blocking appetitive sexual behavior. Thus, infusion of NPY into the brain has a highly specific behavioral effect, changing the rat's preference from a sexual to a food reward (Ammar et al., 2000). Further on, decreasing of the availability of food causes an increase in running, a decrease in eating, and an increase in the synthesis of NPY in the hypothalamus, in a model of food foraging (Nergårdh et al., 2007). Infusion of NPY into the brain replicates the effects on running and eating (Nergårdh et al., 2007).

These results implicate NPY as a mediator of foraging, rather than eating, in an evolutionary conserved neuroendocrine system engaging the NPY Y1 receptor (Lecklin et al., 2003). In the framework of homeostasis, the synthesis of hypothalamic “orexigens” are controlled by leptin and in line with the present perspective, leptin selectively reduces foraging for food in the model discussed above (Ribeiro et al., 2011). This new role of brain messengers, previously thought of as “orexigens,” has been confirmed (Bartness et al., 2011).

Thus, foraging for food increases and eating decreases when food is in short supply (Södersten et al., 2008; Bartness et al., 2011). Conversely, eating without biological constraints is the normal response to an abundance of inexpensive food (Swinburn et al., 2011).

This allows definition of a human homeostatic phenotype, i.e., maintenance of a stable, healthy, low body weight when the physical price of the food is high. As that price approaches zero, eating and body weight increase passively. Most human genotypes allow this development as testified by the marked increase in body weight in many societies, with some variation dependent upon e.g., ethnicity (Ng et al., 2014; Sellayah et al., 2014).

## Anorexia nervosa, cause, and treatment

Anorexia nervosa conforms to the human homeostatic phenotype described above, the high level of physical activity, and the retained capacity to eat a normal amount of food is particularly important in the present context (Södersten et al., 2008). It is long recognized that the “endocrinology of anorexia” is a reversible consequence of starvation (Södersten et al., 2008), and although “an underlying mental disorder” is thought to cause of anorexia (Wierenga et al., 2014), the arguments against this point of view are overwhelming (Bergh et al., 2013). The evidence that any healthy individual will develop *all* symptoms of anorexia nervosa in response to a reduction in food intake is compelling (Keys et al., 1950; Södersten et al., 2008).

Because of the importance of starvation and foraging in human evolution we should therefore “anticipate that genes that enable humans to tolerate prolonged starvation will be found. The search for such genes should, however, probably focus on other phenotypes than anxiety, e.g., those that are capable of a high level of physical activity because of their obvious survival value (Diamond, 2003; Chakravarthy and Booth, 2004). Anorexic patients are likely to have such genes, which should not be labeled ‘disease’ genes” (Södersten et al., 2006). This hypothesis implies that anorexics are resistant to starvation, not that they are in a “normal” condition.

Implemented clinically over the last 20 years, the present perspective relies on a neurobiological framework according to which anorexia nervosa is caused by dieting, activating release of dopamine from limbic terminals of mesencephalic cellbodies, inducing a feeling of “reward,” and encouraging the patient to continue eating less food (Bergh and Södersten, 1996). Dieting also engages the noradrenergic cell bodies of the locus coeruleus, which are part of the brain's system for attention and so anorexia is maintained by conditioning to the cues which provided reward in the first place. Anorexia is the prototypical eating disorder, the other disorders are different expressions of the same problem (Bergh et al., 2002). This framework has been updated (Södersten et al., 2008), including a description of how disordered eating behavior causes mental change (Ioakimidis et al., 2011).

The main intervention is the restoration of normal eating behavior using mealtime visual feedback on a computer screen. The treatment has been described many times, strict criteria of remission must be met, *patients in remission display none of the symptoms diagnosed at admission* (see Bergh et al., 2013). A randomized controlled trial showed that the treatment has a strong effect, the rate of remission was estimated to be 75% in a group of 168 patients, the time to remission was about 12 months and the rate of relapse was about 10% in 83 patients who were followed-up over five years (Bergh et al., 2002). 1428 patients were treated between 1993 and 2011 replicating the same outcome in six different clinics in four countries, and with no mortality (Bergh et al., 2013).

This outcome was obtained despite the fact that 251 patients were severely ill and required in-patient treatment, and despite the fact that the Body Mass Index (BMI) of the 571 anorexic patients (14.9; quartile range 13.8–16.1 kg/m^2^) was very low at admission and normal at remission. This BMI is much lower than the BMI (16.7; SD 0.1) of 242 patients in a recent report (Zipfel et al., 2014). Yet, at 12 months of follow-up, 30% of the patients in that report had dropped out and, although their body weight had increased, none of the patients had a normal BMI (Zipfel et al., 2014).

The same treatment has been adapted for normalizing eating behavior and improving the health of obese children (Ford et al., 2010), supporting the hypothesis that under- and overweight are expressions of the same problem (Bergh et al., 2008).

## Disruption of homeostasis?

The present perspective differs from standard psychiatric approaches in most aspects, and most importantly in outcome. However, it also departs from the theme of this research topic in that anorexia nervosa is considered an example of homeostasis. Thus, food intake and physical activity are in balance, maintaining a constant low body weight. However, anorexics are captured in an undesirable state, unable to escape, they need to re-learn how to eat normally. In this lies the opportunity of developing new methods to treat the patients.

**Figure 1:**
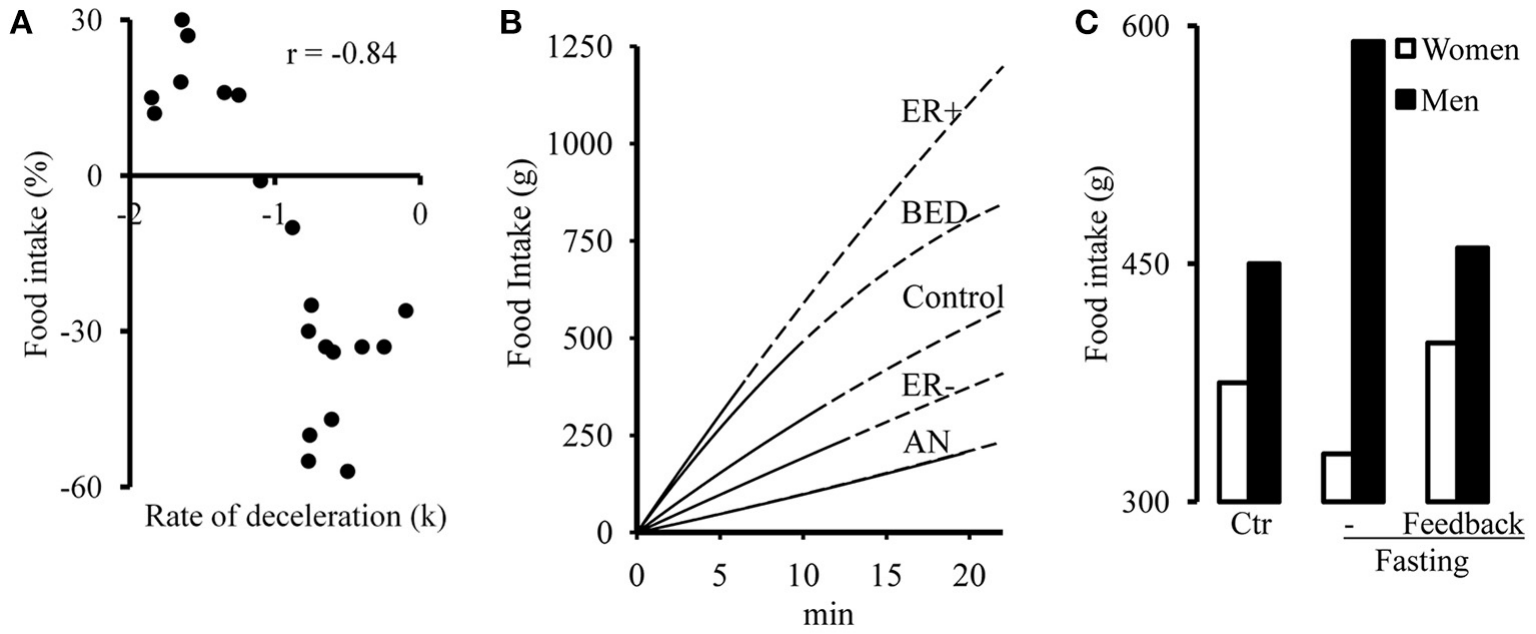
**(A)** Effect of omitted eating dinner on food intake at lunch in 20 women as a function of deceleration (k) of the speed of eating in their un-deprived condition. y = kx^2^ + lx; y = food intake, k = deceleration, i.e., rate at which the speed of eating changes over the course of the meal, l = initial speed of eating and x = time. The *k*-values are from a lunch in the un-deprived condition and the change in food intake is expressed as the percent of that lunch after the women. **(B)** Cumulative food intake in women who ate at a nearly constant rate (k ~ 0, *n* = 16) and female patients with anorexia nervosa (AN, *n* = 16) or Binge Eating Disorder (BED, *n* = 12). The women were challenged to eat a lunch at an increased (ER+) or decreased (ER−) speed. The end of the uninterrupted lines indicates the point in time when the subjects stopped eating; the dashed continuation of the line indicates the extrapolation of the modeled curve. **(C)** Food intake at lunch in 13 women and 9 men in their un-deprived condition (Ctr), after omitting dinner the day before lunch without (− Fasting) or with (− Feedback) visual feedback on how to eat. We thank Elsevier for permission to reproduce **(A,C)** from Zandian et al. (2011) and Springer Science+Business Media for permission to reproduce **(B)** from Ioakimidis et al. (2009).

On the hypothesis that dieting is the main cause for anorexia, it is noteworthy that young women eat less food for lunch after skipping dinner the day before and that k = the rate at which their speed of eating decreases over the course of the meal measured in their pre-deprivation eating behavior determines the size of the effect (Zandian et al., 2011). Food intake is modeled by y = kx^2^ + lx; where y is food intake, k is as just defined, l is the initial speed of eating, and x is time. The more negative the value of k, the better the compensation for a brief period of fasting (Figure 1A). Dieting increases the value of k and as k → 0, i.e., as the speed of eating becomes constant, women are less able to adapt their food intake to challenges such as having to eat slowly, they actually eat less food yet experience a higher level of satiety, thereby approaching the anorexic pattern of eating (Zandian et al., 2009). And when experimentally challenged to eat quickly, they approach the behavior of patients with Binge Eating Disorder (Figure 1B; Ioakimidis et al., 2009). Thus, dieting increases the linearity of eating, exacerbating the risk for losing control over food intake. However, the change in food intake can be reversed by visual feedback on how much to eat over the course of the meal (Figure 1C). Therefore, the biological changes which occur after a brief period of deprivation do not prevent women from maintaining their normal level of food intake (Zandian et al., 2011). Note the marked sex difference in the response to fasting; while women eat less food, men eat more (Zandian et al., 2011), an effect likely related to the fact that 95% of anorexic patients are female.

## Concluding remark

Based on the present perspective, we predicted that the pharmacological project for weight control will fail (Södersten et al., 2006). How could it succeed considering that neural networks permit excessive eating when eating is easy but not when eating requires an effort? A simple change in the environment turns an “orexigen” into an “anorexigen” (Ammar et al., 2000; Nergårdh et al., 2007). From our perspective, eating behavior is the cause of the problems of body weight, not a mere product of the activity of the brain. In support, we found that an experimental change in eating behavior among obese children brought an “orexigen,” ghrelin, under control while improving the health of the children (Ford et al., 2010; Galhardo et al., 2012). This is just one instance of behavioral control of the endocrine system, which is long known in experimental endocrinology (e.g., Lehrman, 1961). We are presently exploring the possibility of using the approach to prevent the problems of body weight.

## Funding

The authors work is supported by Mando Group AB.

### Conflict of interest statement

Södersten and Bergh own 47.5% each of the stock in Mando Group AB, Michael Leon of UC, Irvine, owns 5%. Mando Group AB holds the IPR of Mandometer, the FDA-approved medical device used to treat patients with eating disorders in clinics managed by Mando Group AB. Swedish health care is publically funded. Södersten, Modjtaba Zandian and Ioannis Ioakimidis are appointed by Karolinska Institutet, all salaries are paid by Mando Group AB, Ioakimidis is paid by a EU research grant.
